# The Parasitic Nature of Social AI: Sharing Minds with the Mindless

**DOI:** 10.1007/s12124-020-09523-6

**Published:** 2020-03-17

**Authors:** Henrik Skaug Sætra

**Affiliations:** Østfold University College, Halden, Norway

**Keywords:** Social robots, Artificial intelligence, Cultural psychology, Cooperation, Deception

## Abstract

Can artificial intelligence (AI) develop the potential to be our *partner*, and will we be as sensitive to its social signals as we are to those of human beings? I examine both of these questions and how cultural psychology might add such questions to its research agenda. There are three areas in which I believe there is a need for both a better understanding and added perspective. First, I will present some important concepts and ideas from the world of AI that might be beneficial for pursuing research topics focused on AI within the cultural psychology research agenda. Second, there are some very interesting questions that must be answered with respect to central notions in cultural psychology as these are tested through human interactions with AI. Third, I claim that social robots are *parasitic* to deeply ingrained human social behaviour, in the sense that they exploit and feed upon processes and mechanisms that evolved for purposes that were originally completely alien to human-computer interactions.

## Introduction

Artificially intelligent (AI) machines are capable of many things: they can play games and perform calculations, they can do physical work in factories, they can drive, guide missiles, walk, talk, and much more. However, can they also be our social partners? We have already introduced such machines into our social lives, but how do we perceive these entities – so much alike us in certain aspects, yet also fundamentally different? In this article I examine both of these questions. I agree with Yıldız (2019) that cultural psychology should add such questions to its research agenda, and there are three areas, or research agendas, in which I believe there is need for both a better understanding and an added perspective.

First, I will present some important concepts and ideas from the world of AI that might be beneficial for pursuing research topics focused on AI within the cultural psychology research agenda. Furthermore, it is important to note that how we perceive computers influences how we view human beings and vice versa. However, using human-human interaction to inform the development of AI might strengthen the deceptive potential of AI as discussed in the third part.

Second, there are some important questions that must be answered with respect to central notions in cultural psychology as theories and concepts are tested through human interaction with AI. What happens when you share minds with the mindless, so to speak? Shared intentions, joint meaning, the blurring of boundaries between minds – such concepts have great potential for exploring both the nature of what humans really are and how we interact with each other and machines.

Third, I claim that social robots are *parasitic* to deeply ingrained social behaviour, in the sense that they exploit and feed upon human processes and mechanisms that evolved for purposes that were originally completely alien to human-computer interactions. I examine how this fact might have consequences for how we view computers as tools with regard to Yıldız’s (2019) referential triangle.

I believe that cultural psychology has much to add to the understanding of the nature of our relationship with, and vulnerability to deception by, AI. When self and other “require each other, and dynamically, dialectically, and jointly make each other up”, making robots our *others* could have important consequences (Shweder 1991). Perhaps we will learn more about ourselves when we share minds with robots. That, or a void will be created when we share minds with the mindless. A void we should be wary of.

Yıldız (2019) also argues that understanding human-human interactions, and applying this insight to human-computer interactions, will enable us to make more useful machines. This will, for example, let us build computers from which children can learn *directly*, instead of via the indirect way in which we relate to *things*. Research shows, however, that both young and old interact with very simple robots *as if* they were non-things (Turkle 2011; Darling 2017; Sung et al. 2007). We tend to anthropomorphise things, like when we believe our computer is having a bad day or that our robot vacuum cleaner is upset. Does this proclivity involve an elevation of non-things into something more, or a demotion of ourselves? Depending on our theories on human-human interaction, the answer may be one of, neither, or both of the two options.

## Computers and Our Interactions with Them

### Artificial Intelligence

There are tools, and then there are *tools*. Yıldız (2019) discusses tools in general, and focuses on AI as a particularly interesting tool. Tools are also called *artefacts*, and the devices that “maintain, display or operate upon information” are *cognitive* artifacts, or *psychological tools* (Norman 1991, p. 17; Engeström 2015). AI systems fit well with such a description. These artefacts are important because they have a *mediating* function in human cognition, in addition to the fact that they can *change* the actors that use them (Kaptelinin 1996). See Norman (1991) and Kaptelinin (1992, 1996) for more.

Before I embark upon the question of sharing minds with machines, I wish to emphasise that I focus on a *subset* of what can be labelled AI. If we are to add to and guide the research on AI, we must both be clear about what we mean by AI and what sort of AI research we intend to add to. AI is a label that can be applied to any system capable of performing tasks “commonly thought to require intelligence” (Brundage et al. 2018). As I will return to later, this is not the same as AI having *general* intelligence of the kind discussed by, for example Carroll (1993). AI has achieved great results in very narrow tasks, but is, as of yet, far from achieving high levels of this kind of general intelligence.

Such tasks could be simple assembly line operations or playing intellectually taxing games such as chess and Go. Google’s *DeepMind* is one such system, and while it aims to push the boundaries of AI for “positive impact”, it also has versions such as *AlphaZero* and *AlphaGo* made for playing the aforementioned games (Google 2019a, b). The latest head on the DeepMind medusa is *AlphaStar*, which recently made headlines for achieving the title of grandmaster in the computer game StarCraft II (Google 2019c).

The particular tools we discuss are often described as *learning* through “experience”. This is what we call *machine learning*, and it is closely related to pattern recognition (Bishop 2006). One approach is *reinforcement learning*, where a system is given some ground rules and a goal and then teaches itself the best way to achieve this goal through trial and error (Sutton and Barto 2018). When we provide the machines with human-coded examples to learn from, we call this *supervised learning* (Sutton and Barto 2018). Learning often occurs in what is called *artificial neural networks*, where artificial neurons are connected in reinforcement systems that mimic biological information-processing systems (Bishop 2006). With enough depth, or layers, in these systems, we speak of *deep* learning (Sutton and Barto 2018). *Big data* is not particularly relevant for the questions examined in this article, other than that it often constitutes the experiences we feed our machines in order to make them learn. The science of AI is not particularly new, but due to the massive amounts of data we now have, AI has become far more relevant in many areas (Danaher 2016; Marcus and Davis 2019).

### Social AI and Robots

What sort of AI am I interested in, then? Not the kind that is used by bankers to determine who gets loans or by the legal system to determine who gets bail (Tashea 2018). Neither am I particularly interested in the algorithms that assist me in finding the Netflix content I might enjoy or the Facebook posts that will hold my attention. Bucher (2012, 2018) and Gillespie (2014) provide important accounts of the problems of Facebook algorithms and algorithmic power in general. Gillespie (2010) and Foer (2017) warn us about the role profit-seeking tech companies now have as keepers of culture, while Zuboff (2019) gives a comprehensive account of both the motives and operations relevant in these respects. This is all AI, but not the AI I am interested in.

I am particularly interested in *social* AI – intelligent systems that are made to interact socially with human beings. Yıldız (2019) mentions HAL from the movie *2001: A Space Odyssey* and Samantha in the movie *Her*. These systems are not embodied, and yet they are able to “successfully read intentions, declare intentions, and communicate” (Yıldız 2019).

Even more interesting is perhaps *embodied* AI, which is commonly referred to as *robots*. The term *robot* is notoriously difficult to define, and Gunkel (2018) provides a good account of the various difficulties involved. We might for our purpose rely on Winfield’s (2012) definition of a robot as a device that senses, and purposefully acts in, its environment. A *social* robot, then, can be understood as a robot that interacts socially with human beings. PARO, the robotic seal, is an example of a *social commitment robot* designed to provide *therapeutic* interactions, particularly for the elderly with dementia (Mordoch et al. 2013; Paro Robots, 2019; Wada et al. 2008).

Yıldız (2019) focuses on *learning* and *education*, but I broaden the spotlight to include social relationships in general. This is also the question he asks at several points in his article, when he asks, “can children, for instance, socially interact with artificial intelligence” as they do with other living beings?

### Tools, symbols, and the referential triangle

Social robots are certainly *tools* in some sense, but Yıldız (2019) uses the term *referential triangle* to explain how “human-human relationships are mentally direct”, while our relations with *things* are *indirect*. A *tool* is something to which we have assigned meaning or meanings that are not intrinsic to the thing itself (Searle 1998). People are unique, Yıldız (2019) says, in “inventing and developing tools” and transferring the knowledge of this through signs and culture. He states that tools like books, radios, and TVs are approached in an indirect way in that children require training to make use of these things. The question, then, is do we need to be taught how to use AI, particularly in the form of social robots? If not, what does this imply for our relationship with such tools? Yıldız (2019) himself states that AI is an “active and integrative tool”, which is very different from more static tools like books and television.

The *referential triangle* is a term employed by Tomasello (2009) for describing the relationship that develops between a child, adult, and some object. It is related to the idea of *joint attention* and how human learning is deeply social and reliant on communication and the interplay between persons (or animals or, as we will see, machines). The world we perceive is made up of “at least two individual perspectives”, and the whole is created through joint attention and the dynamics of the referential triangle (Yıldız 2019). We *learn* in the referential triangle, and we have, Yıldız (2019) argues, no direct contact with objects, as “there are always others” involved in such interaction. Children need others in order make use of tools and objects, and Yıldız (2019) focuses on the evolutionary roots of this process.

Yıldız (2019) states that AI still tests “the general idealistic hypotheses of the cognitive-behavioral school”, and that while it has made important advances, it has not achieved human levels of cognition. This is acknowledged by most, and while AI has come far in *narrow* areas, we are nowhere near *broad* – or *general* – intelligence (Marcus and Davis 2019). General intelligence is non-specific intelligence, which means that a machine would be able to do *anything*, and not just, for example, play chess brilliantly (Müller and Bostrom 2014).

While the computational paradigm of the mind allows for symbol *processing*, Yıldız (2019) argues that the *production* of symbols is a uniquely human feature. We “spread social signs”, we “perceive signals in different ways” when they are combined with other stimuli, and we even use these symbols to guide our perceptions of stimuli (Yıldız 2019).

Human culture is based on our symbols, which is what differentiates our cultures from the cultures of other beings (Yıldız 2019). Symbols allow for intergenerational transfer of information, and they are “an expression of the human mental process” (Yıldız 2019). A symbol is, according Yıldız (2019), *social* and points to a “mental representation”. When I communicate a symbol, it is not just a representation of something in my own mind – it is “related to something both in [my] own mind and someone else’s mind” (Yıldız 2019). With symbols, we cross the boundaries of both mind and time.

Here Yıldız (2019) returns to the referential triangle because our relationships with things, tools, *and symbols* are indirect. The reasoning behind this statement is that none of these things initially make sense to a child without the guidance of other persons. Children understand these things as meaningful only in *social interactions*. Without this interaction, there is no meaning. When the meaning is established, the *meaning* is the way through which they approach the symbol or tool (Yıldız 2019).

But what occurs when a social robot – a tool according to Searle’s definition – is perceived as a social partner? When the relationship between the robot and the child is *direct*, without the initial mediation of a more experienced social partner? I return to this point later in the article.

### Understanding AI through understanding humans

Yıldız (2019) states that one of his goals is to contribute to the human-computer interaction literature by discussing human symbol generation and use. This is an important endeavour, and Marcus and Davis (2019) emphasise the need to better understand how humans work in order to enable further progress in AI. In their book *Rebooting AI*, they discuss how modern AI is good at *narrow* tasks, but is still very far from the broad and general intelligence of human beings. Computers can crunch numbers and find correlations, but they cannot *understand* (Marcus and Davis 2019). In this context, Gadamer’s approach to understanding may help us understand why machines struggle with *understanding*. For Gadamer, the concept is *social*, and involves coming to an understanding *with someone* – the term is closely related to *agreement*, and *consent* (Gadamer 2004). Understanding is intimately connected with *language* and social context, and these are challenging phenomena for today’s AI systems.

This is related to an interesting on-going discussion in the philosophy of the social sciences. Computation and correlation are sometimes seen as a form of *explanation* (Zickfeld and Schubert 2019), but according to Malnes (2019) an explanation must also foster *understanding*, which is much more difficult. Malnes (2019) employs the idea of *causation* as a path towards understanding, while Valsiner (2019) proposes *catalysis* as the proper path towards a real understanding of social phenomena. The very ideas of *explanation* and *understanding* are difficult even for *human beings*, and these debates might be beneficial for the work on promoting *understanding* and broad artificial intelligence. One interesting avenue is the attempt to understand everything through the bottom-up approach of neuroscience. In Sætra (2019c), I argue that such an approach to understanding *everything* is hypothetically possible, but nowhere near achievable right now, and that various approaches and levels of explanations must be employed.

Explanation and understanding are important concepts because humans are “compulsive meaning-makers” (Valsiner 2014). If cultural psychology can contribute to such an understanding of how we find and construct meaning, parts of the AI community will be all ears. As discussed by Ibáñez and Cosmelli (2008), many disciplines have moved from the *computational* model of the mind towards fuller cognitive approaches. This implies moving from understanding the mind as a “rule-based, symbol processor” computer to the study of intentionality, intersubjectivity, and ecology of mind (Ibáñez and Cosmelli 2008). Bruner (1990) states that while “cognitive science has made a contribution” with regard to how humans process information, we have gained “technical success at the price of dehumanizing the very concept of mind it has sought to reestablish” (Bruner 1990, pp. 1, 10). He calls for a *renewed* cognitive revolution, with an emphasis on the concept of *meaning* instead of *information processing*, and how “meanings are created and negotiated within a community” (Bruner, p. 11). Kohler (2010) also suggests that seeing humans as machines is based on a flawed understanding of humans, machines, or both.

However, behaviourism and the computational approach to human beings have recently been encouraged by the advent of Big Data (Sætra 2018b; Zuboff 2019). More data are thought to make it possible to truly understand how we function, and the behaviourist and mechanistic approaches to explanation in the social sciences have thus gained prominence (Sætra 2018a, b).

Our metaphors matter, however, and while trying to understand humans through the study of rats involves what Koestler calls ratomorphy, studying humans as if we are similar to computers and robots might constitute *robotomorphy* (Sætra 2018b). I argue that, for example, cognitive and cultural psychology gives important insights into the characteristics of the human mind that are lost if we reduce our scope of research to what a rat, or computer, can do. As such, I encourage moving beyond the idea of the computational mind as discussed by Ibáñez and Cosmelli (2008). Marcus and Davis (2019) encourage this very move from an AI vantage point. The purely computational approach does not merely reduce human beings to something less than human – it even constricts development in AI research.

Another benefit of a better understanding of human-human interactions is that it can inform our development of effective tools. Yıldız (2019) shows how children learn through social interactions, and I argue that we *can* and *do* have social interactions with social robots. If this is accepted, it implies that such robots can be even more effectively employed in learning situations. While I argue that social robots are both parasitic and sycophantic in the way they exploit our social nature, such robots can obviously also exploit our social nature for reasons we accept. My main argument is that we must be aware of how vulnerable we are to social cues and that we must understand that when more and more of our *social* relations consist of social relations with entities without either intentions or minds as we know it, this might have detrimental effects. When we share minds with the mindless, might something get lost in the void?

## Sharing Minds with Robots

As Yıldız (2019) asks whether or not robots *can* be partners, I here argue that they are, in a sense, our partners already. I believe the most important question is what sort of partners they are, and what effects partnering up with them might have on us. Yıldız (2019) cites Okumura et al. (2013), who show that children can share *attention* with robots, but that they still do not learn from this joint attention with robots the same way they do when they join another human in the referential triangle. Research seems to indicate, however, that we *do* relate to machines in more direct and intimate ways than that suggested by Yıldız (2019).

### Robots as Partners

Tools, Yıldız (2019) says, are the by-products of solutions humans produce for social interaction. If so, social robots are certainly a very fitting addition to this list of tools. The main problem, however, is to determine when a tool becomes so sophisticated that it is no longer just a tool – or a *thing* – and instead carries “the potential to be a partner” to its human creators (Yıldız 2019).

The question posed by Yıldız (2019) is: can AI become our partner? He then states that AI *may* develop the potential for partnership, but I will argue that AI already has this potential because of the social nature of humans. Human beings are so eager to make sense of the world, and to develop social relationships, that it really does not take that much for us to consider something a partner. I will here focus mostly on the human party in the relationship, and not on the what characteristics would have to be present in machines for them to be true partners. When I say that robots are already partners, I mean that we already treat them as such, even though they may lack a true capacity for emotion, empathy, trust, respect, and so on.

By this, I emphasise the very important difference between us *perceiving* something as a partner and the ontological traits of objects that make them capable of being *true* partners in some predefined sense. I focus on the first aspect – our perceptions of other things. In ethics, this is what is called the *relational turn* because the relationship we have with something takes precedence over what the object ontologically *is* (Gunkel 2018; Coeckelbergh 2010). While Searle (1997) states that the computer is a useful *tool*, “nothing more nothing less”, Gunkel (2018) provides a thorough overview of the various strong challenges to such a view of AI and social robots.

When people accept robots as companions, we have reached what Turkle (2011) calls the *robotic moment*. In our relations with robots, we are not very concerned about what these entities really *understand* or *know* (Turkle 2011). Thus, the lack of understanding on the part of AI, as described by Marcus and Davis (2019), is not that big of an obstacle to our acceptance of these beings. Neither is the fact that AI is not at a human level of cognition. Furthermore, they also explain our somewhat overdone enthusiasm for the current state of progress in AI as partly based on what they call the *gullibility gap* (Marcus and Davis 2019). Humans have not evolved to distinguish human from machine, “which leaves us easily fooled” (Marcus and Davis 2019). We have evolved to attune ourselves to the minds, intentions, and feelings of others, and we also do this with computers. This is the main topic of this article, and it is a topic on which cultural psychology can shed some light.

Turkle (2011) argues that we *have* reached the robotic moment, and Darling (2016) argues in similar ways that we *do* respond to robots and AI as if they were far more than mere tools and things. I should note that the relations with robots discussed here are not the same as those between a doll and a child. There, the doll is an object upon which we project ourselves, but robots are seen as *subjects* (Turkle 2011). Latikka et al. (2019) examine robot acceptance in general and show that men are more accepting than women, younger people more so than their elders, and that people with more experience with technology are also more prone to accept robots. There are differences between different kinds of robots, however, and it seems that industrial robots are easier to accept than, for example, care robots (Savela et al. 2018; Latikka et al. 2019).

Seeing robots as partners implies that robots become our *social companions*. A companion is here understood to be a social partner that provides more than mere *instrumental* benefits to the other party in the relationship. I could, for example, have a business partner for purely instrumental reasons, but a *companion* is something *more*. Venturing into the dicey waters of definitions, we could say that the human party in the relationship considers the other intrinsically valuable, worthy of respect, morally considerable, etc. This need not be *consciously* acknowledged – it suffices that the companion creates such feelings on *some* level in the human partner (Sætra 2019b).

A robot seal may be introduced to the elderly with dementia for purely instrumental reasons, but if the elderly respond with some kind of affection, the robot will nevertheless become a companion, even if the elderly are able to understand that the robot is just that – a machine (Sætra 2019b). It is not just the elderly who get social machines. Levy (2008) discusses loving robots, and brothels staffed by robots is no longer just a thing of nightmares – or dreams (Scheutz and Arnold 2016; Lockett 2017).

As robots become more and more advanced, they are able to fulfil more and more of our relational needs. We have used robots as companions for a long time, but the scope of our relations with them is ever broadening (Sætra 2019b). It is not simply a question of robots living *up* to human standards, however. One reason for our acceptance of them is that they have qualities that arguably make them *better* than human companions. They are patient, attentive, loyal, and durable (Levy 2008).

Furthermore, a *companion* implies that there is a reciprocity of sorts involved in the relationship. I have mentioned Gadamer’s (2004) view of *understanding* as something inherently social – something involving a meeting of minds. In a relationship with a companion, one might also argue that *dialogue* and mutual accommodation is central. In such a relationship, we might expect the participants to *change* as a result of their interaction. Reaching an *understanding*, for example, tends to imply that the parties involved in some way changed, and reached common ground. In a companionship with a machine, if we agree with Marcus and Davis (2019) that machines are incapable of understanding, only the human changes. The attractivity that Levy (2008) describes is partially due to us not *having* to change much in our relationships with machines. However, as we often do (mistakenly) perceive both agency and intentions from machines, this could lead to unknown changes in the human party in the companionship.

### False Shared Intentionality

Yıldız (2019) asks if the tools can be our partners and if we can interact *directly* with them, as opposed to how we interact with ordinary tools as *things*. He argues that for this to be the case the tool must have a “behavioural system that can read social signals” *and* it must be able to *generate* social signals (Yıldız 2019). He states that AI is *beginning* to do both of these things – but still not *completely*. I believe that Yıldız’s (2019) demands are most likely too strict, as I have shown that we already treat robots, even those with very limited abilities to read and produce signals, as social partners (Armstrong 2013; Sung et al. 2007). Scheutz (2014) states that autonomy combined with mobility creates perceived agency. However, we know that even mobility is not a *necessary* condition for us to perceive agency and mind in AI. Chatbots like ELIZA and various personal assistants, such as Siri, are examples of non-mobile AI (Weizenbaum 1966; Wang 2017).

Yıldız (2019) also asks if AI can “be both a tool and a partner at the same time”. Can children interact with them as they do with human beings, and thus be *two* things in the referential triangle simultaneously (Yıldız 2019)? In Sætra (2019b), I discuss how people have a dual relationship with social robots in that they *consciously* respond to robots as tools, while *unconsciously* responding to them as social partners. This is what I label *partial deception*, which I discuss in the section on AI’s parasitic nature.

The idea of *authenticity* is relevant for the discussion of the relationships we form with machines (Turkle 2007a, 2011). Turkle (2011) asks, “What if a robot companion makes us feel good but leaves us somehow diminished?” Others, like Scheutz (2014) have also warned about the dangers of *unidirectional* emotional bonds between human and robots.

We must understand what happens when the mechanisms that involve the *sharing of minds* are applied by human beings in relationships with entities that have no *minds*. Trevarthen (2012) discusses the theories of Mead and Vygotsky and what occurs when the highly cooperative young *share minds* with each other. It is also what occurs in the referential triangle, where several individual perspectives are joined in a *shared* representation of some phenomenon. Nelson (2010) speaks of the *communities of mind* that become available to children once they develop their social capacities to the degree where they can *share minds and meanings*.

Turkle (2011) argues that the relationships between humans and machines are qualitatively different from those we have with other humans. The *intimacy* and authenticity of human relationships cannot be mimicked by machines, she argues (Turkle 2011). This is supported Damasio (2018), who states that robots have neither *life* nor *feelings*.

But what is the authentic? If humans are nothing more than biochemical machines, soon to be explained by advances in neuroscience, machines *could* have qualitatively similar feelings to us. Metzler et al. (2016) and Cominelli et al. (2018) discuss the possibilities of robots with emotions. Would such machines be able to form *authentic* relationships? While Damasio (2018) seems to have discounted machine authenticity, in Man and Damasio (2019) he co-authors an article describing machines with homeostasis as their basis for feelings. While appearing to be slightly more optimistic about the possibility of feeling machines, they emphasise that these would not be fully equivalent to human feelings (Man and Damasio 2019). It is, however, interesting that Man and Damasio (2019) propose further research on feeling machines and cultural transmission in “societies of homeostatic robots”.

Authenticity could, however, depend on more than the presence of certain physical, physiological or mechanical qualities. Turkle (2007b) thinks that the authenticity of human relationships *cannot* be mimicked, and that machines can be *evocative* but not *authentic*. Dotson (2014) discusses *virtual* others, and also note “how easily social presence and attachment are evoked in human beings”. He then proceeds to state that “[e]mbracing them [virtual others] … carries the risk of an undesirable shift in the collective conception of authentic sociality”; he thus shows how *authenticity* is a social construct, and that virtual others are “postmodern hyperrealities” that has the power to *change* our view of what is authentic (Dotson 2014). Handler (1986) sees “authenticity” as a “cultural construct of the modern Western world”, and notes that it is strongly tied to western *individualism*. It is, he states, about “the part, unit, or individual asserting itself against the rest of the world as a locus of ultimate meaning and reality” (Handler 1986, p. 3). Jones (2010) also connects authenticity to western modernity and “an emphasis on entities and their origins and essences” (Jones 2010). Turkle (2007b, p. 502) states that “traditional notions of authenticity is in crisis”, as indicated by our relationships with machines. She goes on to state that human beings evolved in environments that did not requires us to distinguish between “authentic and simulated relationships”, and she thus clearly connects authenticity to *humanity*, and not to the quality of the relationships itself (Turkle 2007b). She also states that live animals are *authentic*, whereas robots are not (Turkle 2007b, p. 514).

If authenticity is to be a meaningful concept, it must refer to something other than being a human being, or *alive*. I side with Dotson (2014) when he proposes that “reality is meaningfully authentic due to the fact that it is not immediately pliable to one’s whims and desires”, and that “[a]uthentic others demand our engagement according to terms that are defined dialogically, emergent out of the meeting of at least two subjectivities” (Dotson 2014, pp. 16–17). Machines are thus not excluded from forming authentic relationships *per se*, but only insofar as they lack the capacity for dialogical engagement and the meeting of subjectivities.

Another point to note is that if robots *are* able to have authentic relationships, suddenly human beings are little more than the robots and tools that surround us (Sætra 2019a). It is possible to take such a stance, and if we do then human-computer interactions are hardly problematic. If this is all there is, however, much of the cultural psychological research agenda becomes superfluous, so let us assume for now that man is not merely a machine like the robots we make today.

How might we be diminished by relationships with robots? That is the potential danger of the void I discuss later in the article. It is also possible to argue that relationships with objects carry the risk of what Fromm (1994) calls *mental disintegration*. If human beings *need* human relationships, and robots take the place of humans without satisfying the deeper need for human companionship, this could have detrimental effects. Like a drug that induces the feeling of joy, while the person in question is deeply damaged in the process. This might occur if we are, in fact, alone and isolated when we interact with robots.

Even *if* loneliness is conquered by social robots, is that really a good thing? This question is beyond the scope of this article, but writers such as Mill (2004) and Storr (2005) emphasise the importance of *solitude* for mental and societal health. I refer to Turkle (2011) Sætra (2018a) for more extensive accounts of these issues.

## Human Evolution and the Parasitic Nature of Social Robots

### Evolution, Culture, and Our Social Nature

Human behaviour is rooted in biology, Yıldız (2019) writes, and one of our most characteristic behaviours is the herd behaviour that is the foundation of our morality. However, not all behaviour is explained by biology alone. Culture *emerges* from individuals in a way inconsistent with it being solely based on biology (Yıldız 2019). The cultures of any species are *more* than the sum of the behaviour of the herd’s constituent organisms, and culture also influences evolutionary selection (Yıldız 2019).

Our social nature has evolutionary roots. Evolutionary adaptions evolve over a long period of time, and dismissing them when they are no longer suitable *also* takes a long time. This is unfortunate, one might say, if evolved traits now make us respond to, for example, robots in ways we wish we did not. Evolution has rewarded helping behaviour and traits conducive to cooperation (Tomasello 2009), and humanity thus evolved into a highly sociable species, and our young quickly show a tendency to help and assist others as they enter this world (Yıldız 2019).

Whereas evolution is slow, our symbolic abilities have allowed us to quickly build *cultures* where helpful behaviour and cooperation are rewarded (Yıldız 2019; Valsiner 2014). Cultural psychology, which is at the intersection of developmental and social psychology, shows how *culture* shapes behaviour (Valsiner 2014). Culture has the potential to *counteract* or *amplify* evolution and biology, and thus cultural psychology is an important discipline for understanding both our societies and ourselves as individuals (even if the notion of the pure and isolated individual becomes a bit more problematic when we acknowledge that culture is important).

No matter where our proclivities for helping, empathy, and social orientation come from, understanding these proclivities will help us see how we are so easily deceived by things merely *resembling* ourselves and other biotic parts of our environment that we care for, such as animals. Yıldız (2019) calls our minds *radically social*. Understanding this radical sociality has the added benefit of understanding our radical *vulnerability* to deception by things that appeal to this sociality.

### AI’s Parasitic Social Nature

I call AI *parasitic* because it exploits and feasts upon our social nature. AI, and in particular social robots designed with the intention of appealing to this social nature, is purely *sycophantic*. The design and behaviour of such machines are not based on any real desire on the part of machines to be our friends, confidantes, or partners; they are chosen make us respond to the machine in the way the designers desire. I examine the potential negative effects of such deception, but will note that there are also positive effects of robot deception. Ishowo-Oloko et al. (2019) show that there is a trade-off between transparency and efficiency in certain areas of AI, as robots that do *not* disclose their true nature to the human beings they interact with are, for example, able to achieve higher degrees of cooperation in prisoners’ dilemma games played with humans. As discussed in the section of human-computer interaction, robots might actively employ social cues in order to promote learning. Such deception will most likely come with some benefits, but it could still be problematic and undesirable.

Computers often behave in ways that make us attribute life, feelings, intentions, etc., to them. The *as if* performances of machines make them alive *enough* for humans to respond emotionally and bond with them (Turkle 2011). While some may see this tendency as beneficial for the advent of social robotics, I see this tendency as a vulnerability. Sometimes machines are designed to imitate life, but we also anthropomorphise machines that are far from life-like (Sharkey and Sharkey 2012). We even tend to *enjoy* doing so (Sharkey and Sharkey 2011).

The human tendency to overattribute traits of character to machines is, I argue, founded in our deeply social nature. This is a topic on which cultural psychology has important insight to offer. An interesting point in this regard is that Darling et al. (2015) show that we can measure people’s *empathy* by their interactions with lifelike robots. This suggests that the bonds we form with such machines are based on the mechanisms that make us bond with other living things.

I do not examine the *intentions* or *ethics* of the producers of social robots in this article, but merely note that design choices have great consequences for how deceptive robots are and to what degree they prey on our social susceptibilities. One problem for some robots is that people mistreat them. People might play with them, trick them, and toy with them in ways that preclude their intended functioning.

One company in Finland experienced this, and after merely adding a pair of googly eyes to their previously less-than-charming robot, people in its environment (a library) exhibited more positive attitudes and behaviour towards it (Schwab 2019). Disney, and other creators of fictional characters, know all about the tricks to promote a perception of life, such as those described by Johnston and Thomas (1995) in *The Illusion of Life: Disney Animation*. Creators of AI, however, have far more powerful tools in their hands, as their products have both a form of autonomy, mobility, and a physical presence (Scheutz 2014).

I argue that robots have the power to *deceive* us and that the way we respond to social cues make us far more vulnerable to such deception than we would probably prefer. In a study by Bateson et al. (2006), they demonstrate that merely having a poster with eyes on the wall changes people’s behaviour drastically, in a pro-social way. Social cues are important for the deeply social beings called humans.

Before defining two forms of deception, I will mention a famous test described by Turing (2009), aptly named the *Turing test*. Without going into great detail, it involves devising a test in which a human being does not see the entity he interacts with. If the human believes he is *not* interacting with a machine, when he actually is, the machine has passed the test. Ishowo-Oloko et al. (2019) mention how the Google Duplex – a program that can make telephone calls and place restaurant reservations on behalf of its human users – can now pass as a human, and thus pass a basic version of the Turing test. Searle’s *Chinese room* argument revolves around the idea that it is possible to pass a Turing test without any proper *understanding* on behalf of the machine (Searle 1980). I have already discussed the idea of *understanding*, and the fact that computers lack such understanding has little effect on their deceptive powers.

In *First, they came for the old and demented* (Sætra 2019b), I describe how social robots almost invariably involve some form of deception. This deception can be *full*, or it can be *partial*. Full deception occurs when a robot manages to fool a person into believing it is actually real. Deception is achieved on both a conscious *and* unconscious level. Here, a machine actually passes the Turing test of the human involved. While this might seem far-fetched today, we should remember that babies, children, and elderly with dementia, for example, have Turing tests that are perhaps not that hard to pass. If we discuss AI in general, chat-bots and other forms of disembodied AI have great potential for also convincing those of well-functioning minds that they are not machines.

The second form of deception is *partial deception*. This is the form I am most interested in here, and it occurs when a robot manages to elicit emotional and social responses form humans, even if the humans understand on a rational level that they are interacting with a machine. This is the form of deception described by Turkle (2011) and Darling (2016). When we *know* that a machine is not alive, but still hesitate to hurt it, empathise with it, begin to care for it, etc., partial deception is at play. This is related to the idea of “the willing suspension of disbelief” (Jacobsen 1982). However, I argue that it is not always *willing*, in that we are often not aware of the effects such machines have on us, just like the eyes on the poster of Bateson et al. (2006). What I label deception requires that people in some way treat the machines as *subjects*, and not merely as some form of doll.

Danaher (2020) provides a good account of the literature on, and concerns about, robot deception. He discusses three forms of robot deception: *external state deception*, *superficial state deception*, and *hidden state deception*. The first occurs when a robot deceives you about something *external* to the robot. For example, it tells you that it just got news about a major earthquake in Australia, but there was no such news, or earthquake. Lies, in other words. *Superficial state deception* occurs when the robot gives a signal that “suggests it has some capacity or internal state that it actually lacks” (Danaher 2020). Appearing to be *sad* when you say something it recognises as disconcerting, for example. There is no actual sad emotion in the robot, but it emits signals that suggest that there is. This is of interest with regard to autonomy, and agency, as we can *program* robots to appear to be, and even to *claim* to be truly autonomous. If a human being cannot accurately assess the truth of such statements, they involve superficial state deception. The last is *hidden state deception*, which involves emitting, or supressing, signals in order to *conceal* abilities or capacities that it *does* have. This might be the case if we had a social robot that was equipped with the AlphaZero software, making its chess-playing skills superhuman. This robot was made to be a social companion robot, and it was programmed to appear to be *slightly worse* at playing chess than its human owner, *and* to act both frustrated and in awe whenever it lost to its master. The signals would be deceptive both when it came to chess-playing capacity (hidden state) *and* the presence of emotions (superficial state). Likewise, if a robot with emotions *was* made, it could be programmed to hide such emotions, which would be *hidden state deception* (concealing). What I discuss in this article is mainly related to *superficial state deception* (faking), which might lead to both *full* or *partial* deception in my terminology. While Danaher argues that this form of deception is not really deception, I claim that it is, since it involves sending deceptive signals which misrepresent the true character of the machines.

### The Call of the Void

Yıldız (2019) cites research showing that *shared intentionality* is a trait that distinguishes human beings and our “closest evolutionary relatives”. Our social nature is important for most aspects of being human, but Yıldız (2019) emphasises the beneficial effects social interaction has on learning. The modality of stimuli matters, but social interactions seem to matter more (Yıldız 2019).

The uniquely human nature might even make us closer to dogs than genetically more similar species like chimpanzees, as both dogs and infants understand the concept of finger-pointing, for example, while our closer relatives do not (Hare and Tomasello 2005). Whereas at first babies point to what *they* want, they soon point towards what they believe *others* want (Yıldız 2019). When a baby points to a key that it believes its mother wants, we could argue that the baby points to the key not in its own mind, but in its mother’s mind (Yıldız 2019). By such processes we create *dialogic meaning* with others. Even very young children show this behaviour, and it is often spontaneous and unprompted (Yıldız 2019).

What happens when a child is introduced to a social robot? If we program a robot to lose something, and then act as if it searches for it and cannot find it, we can imagine that a child would exhibit the same helpful behaviour. If so, what sort of mind is the baby pointing towards, and what sort of *dialogic meaning* and *shared intentions* exist in this interaction?

The child might perceive this as a real social interaction, but if there is no mind, or intentions – just code – on the part of the robot, what is left is an inauthentic relationship based on deception. Yıldız (2019) cites Bakhtin and Vygotsky when discussing the idea that the representation of things is not formed by direct contact, but through the formation of a dialogic meaning “through the mind of another person”.

Thus we return to the question of what occurs when machines with no minds entice us to attempt to share minds with them anyway. This is the call of the void.

Helping behaviour is exhibited by humans even if no one has *asked* for help; the one in need of assistance may not even be aware of this need, but will still be helped (Yıldız 2019). Children even verbalise their helping behaviour in order to explain their actions and make the recipient of help aware of their need for help (Yıldız 2019). This means that people do not merely understand people’s *intentions* – we are able to autonomously understand people’s *needs*.

The crucial point here, if we exhibit the same behaviour towards machines, is that such understanding is not even closely related to the reading of minds. It is, I argue, based on *introspection* and the assumption that *others are like ourselves* (Sætra 2019b). If so, that explains why a child would help a robot with no mind, intention, or even needs.

Yıldız (2019) refers to Tomasello (2014) and the idea that minds are able to “engage in other minds by interpreting”. This is purportedly the basis of *communication* itself, as it is a symbolic system that allows *interaction* and *synchronisation* between minds. Devin and Alami (2016) relay their work on an implementation of *theory of mind* in robots, which is being done in order to improve human-robot collaborative efforts. Understanding what *others* think, and intend, is important for any collaborative effort. Blum et al. (2018) describe work on similar functionality in robots. In an interview, Winfield, as one of the authors, describes how such a theory of mind might enable robots to understand each other, which takes them “one stop closer to understanding us” (Baraniuk 2018). Gasparyan (2016) argues that structural and post-structural approaches are important for developing a proper theory of mind in AI. This is because *sense* is a fundamental component required both for *understanding* and detecting *semantic environments*, and sense is in the category of “specific human metaphysical intuitions or emotions” (Gasparyan 2016). Structuralism and post-structuralisms definitions of sense make these aspects clear, but *sense* remains a concept that we can “successfully use”, but not *explain* (Gasparyan 2016).

Minds are closed systems, but communication opens them up. When examining the brains of people speaking to each other, mechanisms are at work that makes it difficult to tell the two brains apart because they, in a sense, work in concert (Yıldız 2019). Gunkel (2018) discusses the philosophy of Levinas, and the idea of the *other*, at length in *Robot Rights*. While his agenda is to determine the moral status of machines, I believe it is even harder to argue that machines are equivalent others in the interactional and truly social sense I discuss here than in a moral sense. Gunkel’s (2018) book is highly relevant for Yıldız’s (2019) question of whether or not AI will be welcomed into our moral community, but I here focus on what occurs when we share minds with robots. Evans’s (2012) analysis of the development of the self and our relationships when the *other* is changed – through a symbolic action and action theory framework – is relevant to these questions.

When people engage in *joint attention fields*, they, in a sense, interact with representations in other people’s minds, and this enables the creation of icons and symbols (Yıldız 2019). Culture is thus established by people in collaboration with each other (Valsiner 2014). Culture is a complicated term, and it is sometimes used in problematic ways. In Valsiner (2012, 2014), important accounts of how to best understand the term are provided.

For cultural psychology, there is an important role to be played in understanding cultural differences in how we relate to technology. Wang (2017), for example, shows how what he labels culture affects the tendency to anthropomorphise, and how there are important differences between individualistic and collectivistic approaches to society. Gunkel (2018) also relays some important research on how there are very different approaches to, and perceptions of, human-machine relations in countries like the US and Japan. Jones (2015) and Robertson (2017) are suggestions for further reading on the topic of cultural differences in both our relationships with and production of robots.

Two important questions arise if the mechanisms discussed here are indeed real. First, what happens to people who communicate and interact with machines as if they were real? There is no external mind to synchronise with, but it seems that the same mechanisms are still at play. Second, what happens to our ability to coordinate and interpret each other if we increasingly interact with machines instead of human beings? If the interaction and interpretation of others’ minds are the basis of our abilities to do such things, sharing minds with the mindless might have some effects on how these abilities develop. Furthermore, authentic interaction with others might be the basis of *meaningful experiences* (Nelson 2010).

If sharing minds with robots is the same as sharing minds with humans, all is most likely good. However, it is possible that sharing minds with robots is more akin to achieving joy through medication, or experiencing joy in Nozick’s *experience machine* where everything *seems* real, but nothing really is (Nozick 2013).

## Conclusions

AI has become increasingly social, and in many respects it is already partnering up with human beings. Particularly in the form of social robots. AI means different things, and I argue that it is important that a cultural psychological contribution to the research fields relates to the concepts and developments in other research areas dealing with human-computer interactions as well as human-robot interactions. Yıldız’s (2019) article calls for increased knowledge of human-human interactions in order to better understand human-computer interactions. This is an important research field, and I argue that it might enable progress in the design of both AI in general and robots, and that it might also give us some important answers about how *humans* really function.

I have focused on various aspects of human sociality, and I argue that robots actively exploit the social and helping tendencies in human beings. The fact that we are so easily fooled, and willing to bond, poses a challenge to the social theories of social interaction, such as cultural psychology. These challenges, however, can be positive challenges, in that accepting them might in fact strengthen and improve the discipline.

First, cultural psychologists and others will have to analyse and explain how joint attention, shared intentions, dialogical meaning, and existence in the referential triangle become manifest when people meet machines. If people, as I have shown, interact with machines *as if* they are human, and the machines have neither minds nor intentions, we must explain what exactly is happening. Either the social mechanics described here are quite simply false, and people interact with machines just as they do with humans, *or* we *believe* we interact socially, but something is simply hijacking the evolved processes of social interaction.

That is the second way this challenge can further develop the research field, if we are able to explain how the imaginary sharing of minds affects human beings, our relations, and our culture. As Turkle (2011) proposes, while these relationships with machines *feel* good, they might in fact diminish us.

Cultural psychology has much to offer in the area of human-computer interaction because it involves the study of how “subject and object, self and other … live together, require each other, and dynamically, dialectically, and jointly make each other up” (Shweder 1991). When objects become subjects, and when the others are machines, these questions might require a second look, and knowing the answers becomes urgent.
